# Supplementary material for: The association between hyperlipidemia, lipid-lowering drugs and diabetic peripheral neuropathy in patients with type 2 diabetes mellitus
Source: PLoS One. 2023 Jun 15;18(6):e0287373. doi: 10.1371/journal.pone.0287373 (PMC10270586; doi:10.1371/journal.pone.0287373)
Supplement: Attachment — Submitted filename: Response letter.doc [file pone.0287373.s002.doc]

Dear Editors and Reviewers:

Thank you very much for your kind consideration of the publication of our article entitled “The association between hyperlipidemia, lipid-lowering drugs and diabetic peripheral neuropathy in patients with type 2 diabetes mellitus”. We deeply appreciate the editors’ and reviewers’ valuable comments and have revised our manuscript based on those opinions. For your convenience, the responses to the referees’ comments are shown in red color, and the changes are also shown in red color in the revised version of the manuscript. We would be glad to respond quickly to any further questions or comments.

Best regards,

Ming-Hong Chang, MD

**Answers to Editor’s and Reviewers’ comments**

Reviewer #1: In the present study, the authors analyzed the association between hyperlipidemia, lipid-lowering drugs and diabetic peripheral neuropathy (DPN). Their results showed that neither hyperlipidemia nor lipid-lowering medication was associated with DPN in adults with T2DM. The study population was large and the design was reasonable. But, I still have some concerns to address.

1. The authors analyzed the association of hyper-LDL and hyper-TC with risk of DPN. But why the association of hypertriglyceridemia and low-HDL with DPN were not evaluated. Although the two lipids were not different between two groups, the comparison might be confounded by other factors.

**Ans.** Thank you very much for your kind and useful suggestion to make the manuscript more comprehensive. In univariate analysis, we investigated the association of each lipid profile with the risk of DPN. As shown in Table 1, the baseline and follow-up plasma levels of LDL-C and TG were significantly lower in the DPN group in our study. Meanwhile, regarding plasma levels of HDL-C and TG, there was no difference between patients with and without DPN. Based on these findings, we hypothesized that plasma LDL-C or TC, but not HDL-C or TG, were associated with DPN. Further multivariate analysis was adjusted for all the variables that were significantly different between patients with or without DPN in Table 1. The revision is shown in red color at lines 234-240 in the revised manuscript.

2.Since several studies have shown that hyperlipidemia, including LDL, TG, HDL, were contributors for DPN in T2DM (e.g. a review by Pop-Busui R, et al. Diabetes Care. 2017;40(1):136-54), the authors should discuss more details about the discrepancy (e.g. the differences in examination method, characteristics of subjects, statistical method... )

**Ans.** Thank you very much for your kind and useful suggestion to make the manuscript more comprehensive. We reviewed previous studies that investigated the association of hyperlipidemia and DPN. The comparison between each study is listed below. The limitations of these studies included small sample size, race disparity (mostly Caucasian population), and discrepancy in examination method. The strength of our study included large sample size, standardized data collection, and validated screening tool. The limitation of our study also included race disparity (mostly Han Chinese population). A recent meta-analysis (Zixin Cai et al.) provided similar results with our findings. It implies that with a larger sample size and Han Chinese population included, the association between hyperlipidemia and DPN is not significant. The revision is shown in red color at lines 321-327, 333-336 and 344-345 in the revised manuscript.

- A. Gordon Smith, et al. [1]:
  - Small sample size (218 patients with T2D).
  - All patients were from the USA.
  - The presence of neuropathic symptoms was not strictly required to diagnose DPN. Therefore, the prevalence of DPN could be overestimated.
- K. Van Acker, et al. [2]:
  - 344 patients with T1D and 767 with T2D enrolled.
  - All patients were from Belgium.
  - Only patients with painful DPN had hyper-TG. When it comes to all patients with DPN, neither hyper-TG nor hyper-TC was associated with the risk of DPN.
- Solomon Tesfaye, et al. [3]:
  - Only patients with T1D were enrolled.
  - The patients were from European countries.
- Johann M. E. Jende, et al. [4]:
  - Small sample size (100 patients with T2D).
  - The patients were from Germany.
  - The diagnosis of DPN relied on the neuropathy symptoms score (NSS) and the neuropathy deficit score (NDS). NSS was not designed specifically for DPN evaluation, and it was found that NSS does not reflect DPN progress. Moreover, some elements of NDS have not been essentially abnormal in DPN. [5]
  - The result mentioned that hypercholesterolemia, including hyper-TC and hyper-LDL-C, was negatively associated with DPN.

| Studies | Findings | Size | Region / Countries | Characteristics of subjects with DPN | Exam methods | DPN definition |
| --- | --- | --- | --- | --- | --- | --- |
| Our study | Hyperlipidemia is not associated with DPN in patients with T2D. | 2448 T2D | Taiwan | ○ Age: 71.1±12.4  ○ 524 (21.4%) had DPN  ○ HbA1c: 7.5 ± 1.5  ○ Diabetes duration: 13.9 ± 9.4 years | ○ Michigan neuropathy screening instrument (MNSI) | ○ ≥ 2 in MNSI |
| A. Gordon Smith, et al. (2013) [1] | Hyper-TG increased risk for DPN  (mainly for small fiber neuropathy). | 218 T2D | USA | ○ Age: 60 ± 7.9  ○ 33 (15%) had no neuropathy, 101 (47%) probable neuropathy, 83 (38%) possible neuropathy  ○ HbA1c: 7.2 ± 1.9  ○ Diabetes duration: 8.67 ± 6.50 years | 6 Criteria of DPN:  Symptoms, signs (Utah Early Neuropathy Scale), NCS, QST, QSART, measurement of IENFD | ○ Probable DPN: ≥ 3/6 criteria  ○ Positive DPN: 1 or 2/6 criteria |
| K. Van Acker, et al. (2009) [2] | DPN was only associated with low HDL level, but not with high TG or TC. Patients with painful DPN had higher TG and lower HDL levels. | 1111 (767 T2D, 344 T1D) | Belgium | ○ Age: 45.9 ± 15 (T1D), 63.6 ± 11 (T2D)  ○ 25.6% (T1D); 50.8% (T2D) had DPN  ○ HbA1c: 7.8 ± 1.17 (T1D); 7.58 ± 1.29 (T2D)  ○ Diabetes duration: 16.5 (T1D), 11 (T2D) | Neuropen | ○ DPN: positive Neuropen test  ○ Painful DPN: positive Neuropen test + ≥ 4 in DN4 questionnaire |
| Solomon Tesfaye, et al. (2005) [3] | High TG, TC, and LDL were associated with DPN. | 1172 T1D | 31 diabetes clinics across Europe | ○ Age: 30.7 ± 8.8  ○ 276 (23.5%) patients had DPN  ○ HbA1c: 8.4 ± 1.9  ○ Diabetes duration: 11.6 ± 7.6 years | Clinical evaluation and physical examination | ≥ 2/4 criteria:  ○ Symptoms  ○ Areflexia  ○ Abnormal sense of vibration  ○ Dysautonomia |
| Johann M. E. Jende, et al. (2019) [4] | Low TC, HDL, and LDL were associated with more lipid equivalent lesion (LEL). | 100 T2D | Germany | ○ Age: 67.49 ± 1.09  ○ 64 with DPN, 36 without DPN  ○ HbA1c: 7.40 ± 0.54  ○ Diabetes duration: 12.81 ± 1.35 years | MRN (magnetic resonance  neurography) | ≥ 5 in NSS or NDS + abnormal NCS in two different nerves |
| Zixin Cai, et al. (2021) [6] | TC, LDL, HDL, TG levels made no significant difference with the risk of DPN in T2D patients. | Meta-analysis | 39 studies from Asia, Europe, North America |  |  |  |

MNSI: Michigan neuropathy screening instrument; NCS: nerve conduction studies; QST: quantitative sensory testing; QSART: quantitative sudomotor reflex testing; IENFD: intraepidermal nerve fiber density; DP4: Douleur Neuropathique 4 Questions; LEL: lipid equivalent lesion; MRN: magnetic resonance neurography; NSS: neuropathy symptom score; NDS: neuropathy deficit score

3. In the multi-regression analysis, some factors were adjusted (Table 2, 3, 4). It should be explained why these factors were selected to adjust. Especially, how to adjust the type of diabetes treatment? I think the table and legends could be more detailed.

**Ans.** Thank you very much for your kind and useful suggestion to make the manuscript more comprehensive. The table legends and results section are revised to include more details regarding the covariate adjustment. The revision is shown in red color at lines 211-217, 229-230, 261-262, and 282-283 in the revised manuscript.

4. How was the sample size calculated?

**Ans.** Thank you very much for your precious comments. In this study, we enrolled the patients that fulfilled the inclusion criteria, which constituted our sample size. We believe that with this sample size, our results are convincing.

5. How about the liver and renal functions (e.g. ALT, AST, Cr) in each group? Whether the patients with severe abnormal liver or renal functions were excluded, since the abnormal function could affect the blood lipid levels.

**Ans.** Thank you very much for your precious comments. In this study, liver function was assessed by liver disease, based on ICD-9-CM 571 to 573. Renal function was measured by estimated glomerular filtration rate (eGFR). The prevalence of liver disease showed no difference between patients without or with DPN, as shown in Table 1. Patients with DPN had significantly lower eGFR than those without DPN. We did not exclude those patients who had severe abnormal renal functions; instead, eGFR was adjusted in the multivariate regression analysis.

Reviewer #2: # Review
The manuscript is written clearly. A large sample size is included. A thorough statistical analysis is performed. The data are clearly presented and justify the conclusions. The references are up-to-date.

**Ans.** Thank you so much for your kind comments. We are so grateful.

**Reference**

1.	Smith AG, Singleton JR. Obesity and hyperlipidemia are risk factors for early diabetic neuropathy. J Diabetes Complications. 2013;27(5):436-42. Epub 2013/06/05. doi: 10.1016/j.jdiacomp.2013.04.003. PubMed PMID: 23731827; PubMed Central PMCID: PMCPMC3766404.

2.	Van Acker K, Bouhassira D, De Bacquer D, Weiss S, Matthys K, Raemen H, et al. Prevalence and impact on quality of life of peripheral neuropathy with or without neuropathic pain in type 1 and type 2 diabetic patients attending hospital outpatients clinics. Diabetes Metab. 2009;35(3):206-13. Epub 20090317. doi: 10.1016/j.diabet.2008.11.004. PubMed PMID: 19297223.

3.	Tesfaye S, Chaturvedi N, Eaton SE, Ward JD, Manes C, Ionescu-Tirgoviste C, et al. Vascular risk factors and diabetic neuropathy. N Engl J Med. 2005;352(4):341-50. doi: 10.1056/NEJMoa032782. PubMed PMID: 15673800.

4.	Jende JME, Groener JB, Rother C, Kender Z, Hahn A, Hilgenfeld T, et al. Association of Serum Cholesterol Levels With Peripheral Nerve Damage in Patients With Type 2 Diabetes. JAMA Netw Open. 2019;2(5):e194798. Epub 2019/06/01. doi: 10.1001/jamanetworkopen.2019.4798. PubMed PMID: 31150078; PubMed Central PMCID: PMCPMC6547108.

5.	Yang Z, Chen R, Zhang Y, Huang Y, Hong T, Sun F, et al. Scoring systems to screen for diabetic peripheral neuropathy. Cochrane Database of Systematic Reviews. 2018;(7). doi: 10.1002/14651858.CD010974.pub2. PubMed PMID: CD010974.

6.	Cai Z, Yang Y, Zhang J. A systematic review and meta-analysis of the serum lipid profile in prediction of diabetic neuropathy. Sci Rep. 2021;11(1):499. Epub 2021/01/14. doi: 10.1038/s41598-020-79276-0. PubMed PMID: 33436718; PubMed Central PMCID: PMCPMC7804465.
